# Atlanta Medical College

**Published:** 1868

**Authors:** 


### ATLANTA MEDICAL COLLEGE.

The exercises of this Institution commenced at the appointed time, and are progressing regularly. A modification of the curriculum of study, so as to make "Clinical Medicine" a regular Chair in the College, adds greatly to the interest of the Course, with those soon to assume the practical duties of the profession. An hour and a half every day devoted in this way, with the amount of material at the command of the Professor of this Department, could not be more profitably employed. More than this, and the Surgical Clinic once or twice a week, however, cannot well be spared from the investigation of the fundamental branches of medicine, by those even who are in their second or third course of Lectures, but have not yet graduated.

The class in attendance is much larger than any since the war; and the young men composing it compare favorably with those of any medical class we have ever seen. With the energy and determination exhibited on the part of the Faculty, and the promptness and close attention given by all the students, the present session will doubtless be made the most successful one ever held in the institution.

We sincerely deplore the sad affliction of Prof. Means' family, by which he has been deprived the pleasure of meeting the class at his regular hour on a few occasions. Aside from this melancholy circumstance, no want of regularity and harmony is found to interfere with the satisfactory and successful progress of the course of instruction.

